# A peek into the pipeline: Expert views on emerging therapies

**DOI:** 10.1016/j.xcrm.2025.102083

**Published:** 2025-04-15

**Authors:** Ilse Dewachter, Günter U. Höglinger, Daniel J. Drucker, Christine E. Brown, Roland Martin, Anne Bernadette Chang, Sanjay Haresh Chotirmall, Xiaolong Qi, Pamela Shaw

## Main text

### Alzheimer’s disease (AD) trials: A field in (r)evolution

**Figure:**
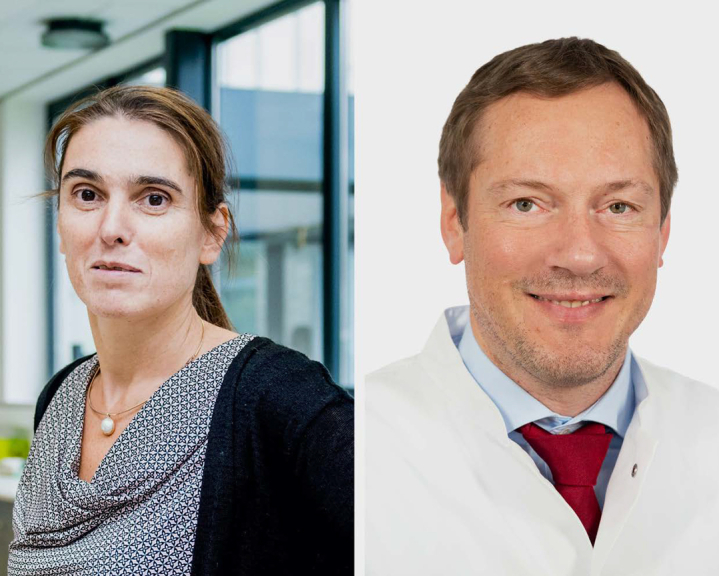
Ilse Dewachter and Günter U. Höglinger BIOMED and Hasselt University University Hospital of Munich, German Center for Neurodegenerative Diseases (DZNE)

AD remains a major societal challenge, but more than a century of research is gradually leading to an exciting (r)evolution in AD and other tauopathy trials. Initially, amyloid-beta-targeting trials faced significant failures, but recent successes with lecanemab (Clarity AD) and donanemab (TRAILBLAZER-ALZ 2) demonstrated substantial amyloid clearance and significant clinical benefits. These breakthroughs marked the first disease-modifying effects in AD trials, leading to FDA approval (Leqembi in 2023; Kisunla in 2024). While available in the US, China, and Japan, Europe has yet to approve them.

Acknowledging the need for more effective disease-modifying therapies, research is including tau-targeting treatments. First-generation tau immunotherapies failed to meet primary endpoints, but next-generation approaches may show promise (CTAD 2024). Bepranemab (TOGETHER, MTBR-tau immunotherapy) significantly slowed tau pathology progression, as measured by PET, for the first time (Alzforum). While the primary endpoint (CDR-SB) was not met, subtle cognitive improvements (ADAS-cog-14) were observed in predefined subgroups with low tau and non-ApoE4 carriers. Antisense oligonucleotide therapy (e.g., BIIB080, NIO752) is also emerging as a promising tau-targeting strategy for slowing tau pathology and symptom progression. This (r)evolution in tau therapies and their outcomes provides a foundation for developing next-generation, more effective tau therapies, possibly in combination with anti-amyloid treatments.

Beyond amyloid and tau, new targets, like inflammation, ApoE, and TREM2, are under investigation. Biomarker research has (r)evolved from amyloid-/tau-CSF to amyloid-/tau-PET and recently to blood-based biomarkers. Clinical trial designs have also transformed, accelerating the development of disease-modifying treatments. These advances provide a solid foundation for developing more-effective therapies.

### The cardiorenal benefits of glucagon-like peptide 1 (GLP-1) medicines

**Figure:**
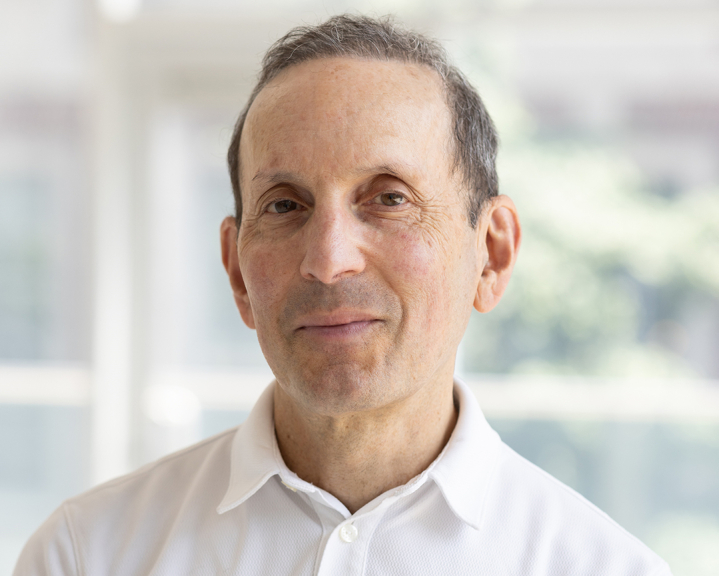
Daniel J. Drucker Lunenfeld-Tanenbaum Research Institute, Mt. Sinai Hospital and University of Toronto

Initially developed as glucose-lowering medicines for type 2 diabetes (T2D), GLP-1 therapies were subsequently approved for weight loss over a decade ago. The requirement for cardiovascular safety trials for T2D therapies ushered in a new evidence-based era for GLP-1 medicines, demonstrating a reduction in myocardial infarction, stroke, cardiovascular death, and all-cause mortality in people with T2D treated with long-acting GLP-1 medicines. More recently, the cardiorenal benefits of semaglutide were extended to encompass a 24% reduction in rates of progression to chronic kidney disease and cardiovascular death in the FLOW trial, a dedicated renal outcomes trial studying semaglutide 1 mg once weekly in people with T2D. Equally exciting are the results of the SELECT trial studying the safety of semaglutide 2.4 mg once weekly in subjects with overweight or obesity and a history of atheroscleoritc cardiovascular disease. Semaglutide reduced the rate of the composite primary outcome, myocardial infarction, stroke, and cardiovascular death, by 20%, associated with a 19% reduction in all cause mortality. Notably, scrutiny of adverse events across the FLOW and SELECT trials revealed a favorable benefit-risk profile of semaglutide therapy, without evidence for new concerning adverse events. These two landmark trials continue a promising shift away from a historical glucose- or weight loss-centric focus in the treatment of T2D and obesity, respectively, further emphasizing the substantial benefits of GLP-1 medicines in reducing serious cardiorenal complications, morbity, and mortality in people living with T2D and/or obesity.

### The promise of chimeric antigen receptor (CAR) T cells in glioblastoma

**Figure:**
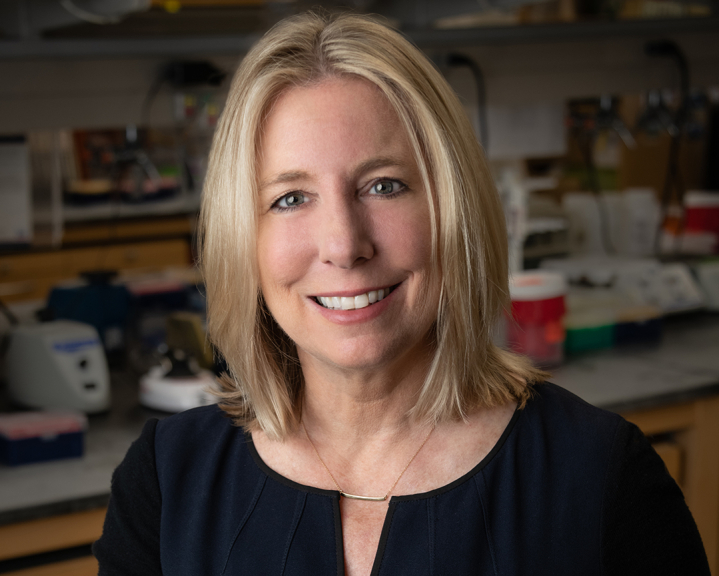
Christine E. Brown City of Hope Beckman Research Institute and Medical Center

My phone rang. “All the tumors have disappeared,” said my collaborator, Dr. Behnam Badie, the lead clinical investigator of our newly opened CAR T cell trial for glioblastoma after treating our first patient. Was this possible? One of the most aggressive and treatment-resistant tumors—a recurrent, multifocal glioblastoma—had melted away after several infusions of CAR T cells, delivered for the first time directly into the patient’s cerebrospinal fluid.

Fast forward ten years with experience from our institution and other centers, responses like this one and the all-too-frequent therapeutic failures have highlighted the potential of CAR T cell therapy for malignant brain tumors and the critical challenges that remain. The field has largely embraced locoregional delivery to the cerebrospinal fluid to bypass the blood-brain-barrier and improve CAR T cell penetration into tumors. We now understand that CAR T cells profoundly alter the immune landscape within the brain with evidence for IFN-pathway cytokines being associated with therapeutic responses. Clinical insights have also identified suppressive pathways, such as TGF-β, that limit CAR T cell responses. We and others have demonstrated that a broad range of tumor-associated antigens can be safely targeted, including IL-13Rα2, HER2, EGFR, GD2, and B7H3, and new trials exploring multi-antigen strategies are yielding promising results.

These learnings are driving innovative approaches to tackle the key challenges associated with glioblastoma immunotherapy. These next-generation approaches, including enhancements to CAR T cell fitness, immune modulation, and combinatorial strategies, hold the key to making durable responses a reality for more patients.

### Autoimmune diseases: The challenge of re-inducing tolerance

**Figure:**
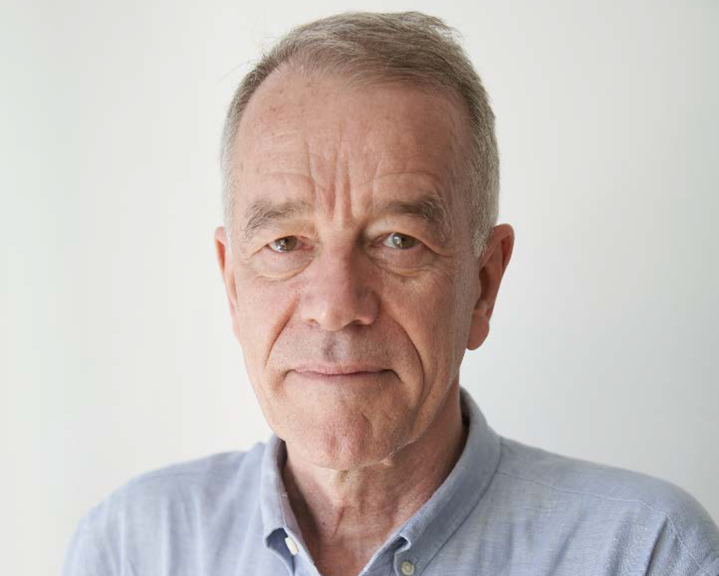
Roland Martin University of Zurich, Cellerys AG, and Karolinska Institute

Autoimmune diseases (AIDs) like type 1 diabetes (T1D) and multiple sclerosis (MS) are caused by aberrant immune responses targeting self-antigens of specific tissues. AIDs are treated with various immunomodulatory/-suppressive drugs. Despite improvements in efficacy, their side effect profile remains suboptimal. For over three decades, immunologists have pursued re-inducing immune tolerance through reverse vaccination, silencing the autoimmune response in a precise and antigen-specific manner. Tolerance induction involves delivering the target antigens as self-peptides or -proteins in a tolerogenic rather than immunogenic way. This works convincingly in animal models by injection of antigen-coupled cells, encapsulation in or coupling onto nanoparticles, or adding a targeting moiety for delivery to specialized antigen-presenting cells (APCs) in organs like the liver and spleen. However, translation to humans has been much more difficult, with most early- and late-stage clinical trials essentially failing. In the past decade, several companies and academic groups have intensified their efforts, and we expect promising developments from phase Ib/II trials soon (e.g., NCT04602390/ANK-700 and NCT06430671/CLS12311 in MS; NCT05104853/CNP-104 in primary biliary cholangitis). Challenges remain in identifying target antigens in specific AIDs, understanding tolerance mechanisms in humans, detecting rare autoreactive T cells, and, last but not least, developing suitable outcome measures and trial designs for early phase clinical trials. There is, however, optimism that this field is moving forward and if finally successful it will become a major breakthrough in medicine.

### Voices of hope on the horizon for bronchiectasis

**Figure:**
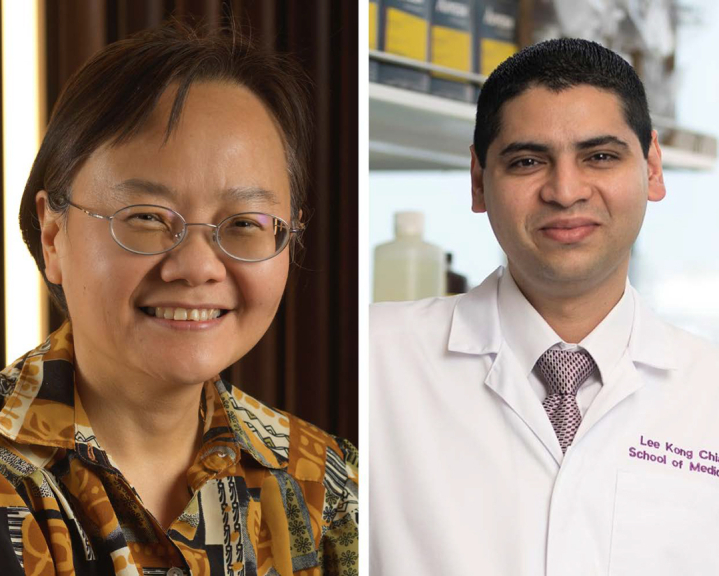
Anne Bernadette Chang and Sanjay Haresh Chotirmall Queensland University of Technology Lee Kong Chian School of Medicine, Nanyang Technological University

Until now, and despite the high burden and cost of bronchiectasis to patients, their families, and health system, no bronchiectasis-specific-approved medications are currently available. There is new hope as the FDA is considering the approval of brensocatib, a reversible inhibitor of dipeptidylpeptidase 1 (DPP1).

The ASPEN study showed that patients receiving brensocatib for 52 weeks (vs. placebo) had a lower annualized rate of pulmonary exacerbations and increased odds of remaining exacerbation free. Those on the higher dose (25 mg) also demonstrated significantly lower lung function decline (forced expiratory volume over 1 s).

In the pipeline, other pharmaceutical companies are examining the efficacy of other agents with comparable mechanisms. DPP1 inhibitors are efficacious through their impact on reducing the activity of neutrophil serine proteases (NSP) including neutrophil elastase (NE), cathepsin G, and proteinase 3. A key mechanism contributing to the pathobiology of Bronchiectasis is the increased accumulation and/or activation of airway neutrophils, leading to NSP dysregulation.

Further, brensocatib (and likely other DPP1 inhibitors) have anti-inflammatory effects extending beyond NSP inhibition including blunting other detrimental effects of NE on host defenses, e.g., the pro-inflammatory mucin MUC5AC. While the relative efficacy of DPP1 inhibitors vs. antibiotics (e.g., macrolides) in reducing exacerbations remain unclear, we welcome its emergence as a treatment option for adults with bronchiectasis and await data on its impact in pediatric populations.

### New treatments and prognostics for chronic liver diseases

**Figure:**
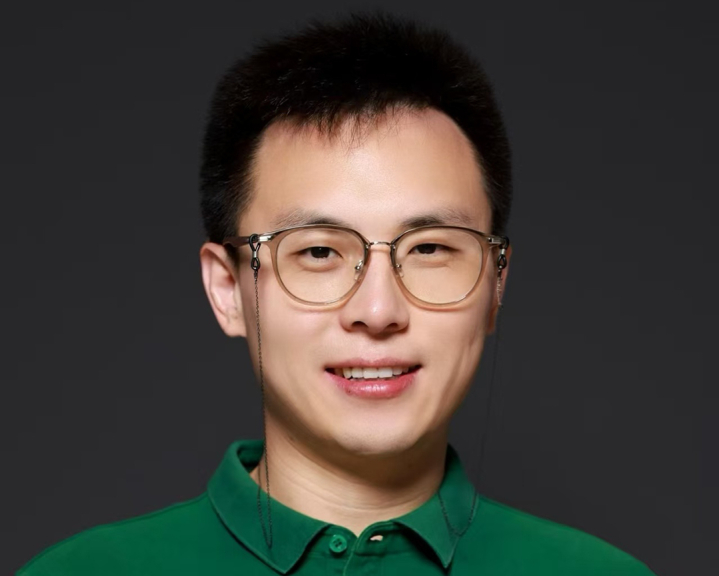
Xiaolong Qi Liver Disease Center, Zhongda Hospital, Southeast University

The approval of resmetirom for metabolic dysfunction-associated steatotic liver disease (MASLD) represents a milestone in metabolic liver disease treatment. However, effective management extends beyond pharmacotherapy, as precise risk stratification is essential for guiding treatment decisions and optimizing clinical trial design. Noninvasive assessments, such as liver stiffness measured by vibration-controlled transient elastography, have demonstrated superior prognostic accuracy.

Building on this, the Baveno VII criteria have redefined the non-invasive diagnosis of clinically significant portal hypertension (CSPH), which drives initial liver decompensation and is marked by a hepatic venous pressure gradient (HVPG) of ≥10 mmHg. The criteria incorporate liver stiffness as a key tool, thereby reducing the need for invasive and expensive HVPG measurements. Whether such non-invasive approach can also effectively guide pharmacological treatment decisions remains uncertain. Non-selective beta blockers, which lower portal pressure by decreasing portal venous inflow, have been proven effective in preventing hepatic events in patients with cirrhosis and CSPH. In this context, the Liver Health Consortium in China (CHESS) recently conducted a randomized, double-blind, placebo-controlled, multicenter trial that validated the effectiveness of the Baveno VII non-invasive criteria for guiding beta blocker treatment. Specifically, this trial evaluated the effect of carvedilol in preventing cirrhosis decompensation in patients with CSPH stratified by liver stiffness. Nevertheless, the effectiveness of other treatments, such as physical activity and statins, in preventing hepatic decompensation should be further confirmed in patients with MASLD-related cirrhosis and CSPH.

### Exciting progress for amyotrophic lateral sclerosis (ALS)/motor neuron disease (MND) therapy

**Figure:**
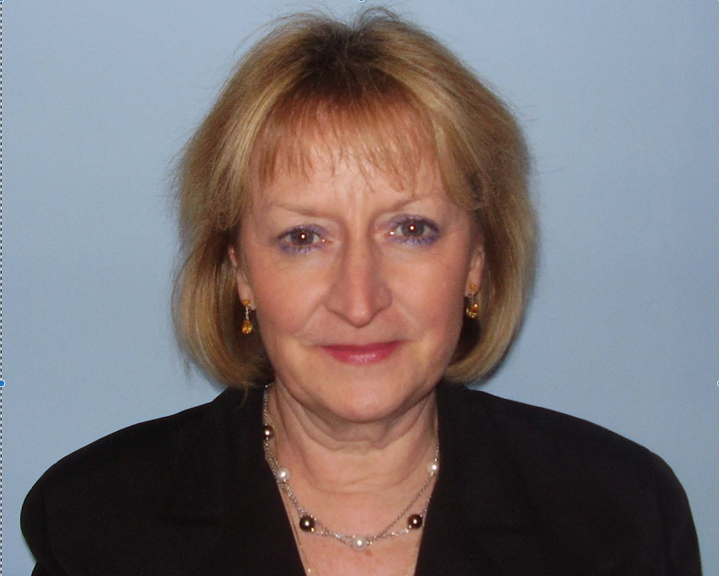
Pamela Shaw Sheffield Institute for Translational Neuroscience, University of Sheffield

In 2023, tofersen became the first approved genetic therapy for 1 of >30 ALS subtypes, lowering the target SOD1 protein and neurofilament levels (NFs) 6 months before unprecedented clinical improvements in the trial. This validated NFs as early biomarkers for motor neuron protection, now embedded as a rapid objective readout for clinical trials.

Recently, jacifusen, another antisense oligonucleotide (ASO), has been shown to reduce FUS (a protein linked to another ALS subset) in a single patient and is now being evaluated with NFs and clinical assessment at phase 3. In contrast, repeat-expansion-targeted trials (BIIB078 and WVE-004 for C9orf72-ALS and BIIB105 for ATXN-2) had no effect or elevated NFs, with no clinical benefits, despite target proteins being lowered in cerebrospinal fluid, so were discontinued.

Therapeutic strategies for sporadic ALS relevant to all subtypes are being screened by platform trials like HEALEY, EXPERTS-ALS, and MND-SMART assessing multiple treatments simultaneously in small cohorts. Platforms can now use NFs to progress candidate therapeutics to phase 3 or be withdrawn for futility, allowing evaluation to progress much faster.

Repurposed compounds are the first arms of platform trials while novel approaches are also emerging. A NRF2 and HSF1 activator, M102, targeting multiple pathophysiological mechanisms contributing to motor neuron injury, is entering first-in-human trials. Low-dose interleukin-2 was safe, well tolerated, and increased regulatory T cells in the MIROCALS trial with a survival benefit in patients with slow to moderate disease progression. Baseline NFs can now predict progression rate, a step change in patient stratification.

## Declaration of interests

G.U.H. has ongoing research collaborations with Roche, UCB, and Abbvie; serves as a consultant for Abbvie, Alzprotect, Amylyx, Aprinoia, Asceneuron, Bayer, Bial, Biogen, Biohaven, Epidarex, Ferrer, Kyowa Kirin, Lundbeck, Novartis, Retrotope, Roche, Sanofi, Servier, Takeda, Teva, and UCB; and received honoraria for scientific presentations from Abbvie, Bayer, Bial, Biogen, Bristol Myers Squibb, Esteve, Kyowa Kirin, Pfizer, Roche, Teva, UCB, and Zambon. D.J.D. has served as a consultant within the past 12 months to Amgen, AstraZeneca, Insulet, Kallyope, and Pfizer and as a speaker for Boehringer Ingelheim and Novo Nordisk Inc. Research in the Drucker lab is funded in part by investigator-initiated grants from Amgen, Eli Lilly, Novo Norfisk, Pfizer, and Zealand Pharma. D.J.D. holds non-exercised options in Kallyope. C.E.B. has patents related to CAR T cells and their delivery for the treatment of brain tumors. R.M. has received unrestricted grants from Biogen, Novartis, Roche, and Third Rock and honoraries for advisory roles and lectures from Roche, Novartis, Biogen, Genzyme, Neuway, CellProtect, Third Rock, Teva, and Swissrockets. He is a patent holder and co-holder on patents of daclizumab in MS (held by NIH) and JCV VP1 for vaccination against PML, JCV-specific neutralizing antibodies to treat PML, antigen-specific tolerization with peptide-coupled cells, novel autoantigens in MS, and designer neoantigens for tumor vaccination (all held by University of Zurich). He is a co-founder of Abata Therapeutics, Watertown, MA, USA, and co-founder and employee of Cellerys AG, Schlieren, Switzerland. A.B.C. has served on the advisory board for Boehringer Ingelheim and on Data Safety and Monitoring Boards (DSMB) for AstraZeneca, Moderna, and GSK, all outside of the submitted work, with monies received by her institution. She serves on the editorial boards of Cochrane, *Lancet Respiratory Medicine*, *Pediatric Pulmonology*, and *Journal of Clinical Medicine*. S.H.C. has served on advisory boards for CSL Behring, Pneumagen Ltd., Sanofi, Boehringer Ingelheim, and Zaccha Pte. Ltd, has served on Data Safety and Monitoring Boards (DSMB) for Inovio Pharmaceuticals and Imam Abdulrahman Bin Faisal University and received lecture fees from Astra-Zeneca and Chiesi Farmaceutici, all outside of the submitted work. He serves as Deputy Editor of the *American Journal of Respiratory and Critical Care Medicine* (AJRCCM). P.S. was the European Chief Investigator for the Phase1.2.3 trials of tofersen, sponsored by Biogen.

